# Sinonasal adenoid cystic carcinomas accompanied by seromucinous hamartoma and/or atypical sinonasal glands arising from seromucinous hamartoma: insight into their histogenesis

**DOI:** 10.1007/s00428-025-04053-1

**Published:** 2025-02-22

**Authors:** Martina Bradová, Abbas Agaimy, Jan Laco, Petr Martínek, Stanislav Kormunda Ing, Cécile Badoual, Ivan Damjanov, Ilmo Leivo, Carlos E. Bacchi, Eva Comperat, Stephan Ihrler, Niels J. Rupp, Radek Šíma, Petr Šteiner, Tomáš Vaněček, Sarina Mueller, Sami Ventelä, Alena Skálová, Michal Michal

**Affiliations:** 1 Sikl’s , Department of Pathology, Charles University, Faculty of Medicine in Plzen, E. Benese 13, 305 99 Pilsen, Czech Republic; 2 Bioptic Laboratory, Ltd, Plzen, Czech Republic; 3 Institute of Pathology, University Hospital Erlangen, Friedrich‐Alexander University , Erlangen‐Nürnberg (FAU), Comprehensive Cancer Center (CCC) Erlangen-EMN, Erlangen, Germany; 4 The Fingerland Department of Pathology, Charles University, Faculty of Medicine and , University Hospital Hradec Kralove, Czechia, Czech Republic; 5 Molecular and Genetic Laboratory, Bioptic Laboratory, Ltd, Plzen, Czech Republic; 6 Service d’Anatomo-Pathologie, Department of Pathology, Hôpital Européen G Pompidou, APHP, Université de Paris, 20-40 Rue Leblanc, 75015 Paris, France; 7 Department of Pathology and Laboratory Medicine, The University of Kansas School of Medicine, Kansas City, KS USA; 8 Institute of Biomedicine, Pathology, University of Turku, Turku, Finland; 9 Department of Pathology, Turku University Hospital, Turku, Finland; 10 Consultoria Em Patologia, Botucatu, São Paulo, Brazil; 11 Department of Pathology, Tenon Hospital, Sorbonne University, Paris, France; 12 Department of Pathology, Medical University of Vienna, Vienna, Austria; 13 Dermpath, Muenchen, Germany; 14 Department of Pathology, and Molecular Pathology, University Hospital Zurich, Zurich, Switzerland; 15 Department of Otorhinolaryngology and Head and Neck Surgery, University Hospital Erlangen, Erlangen, Germany; 16 Department of Otorhinolaryngology, Turku University Hospital, Turku, Finland

**Keywords:** Adenoid cystic carcinoma, Respiratory epithelial adenomatoid hamartoma, Seromucinous hamartoma, MYB, MYBL1, NFIB, Atypical sinonasal glands arising in seromucinous hamartoma

## Abstract

The pathology of reactive, dysplastic, and neoplastic sinonasal seromucinous glands is complex, and their contribution to tumorigenesis of sinonasal carcinomas remains controversial. In our practice, we have observed the presence of respiratory epithelial adenomatoid hamartomas (REAH) and seromucinous hamartomas (SH) associated with adenoid cystic carcinomas (AdCC) in a subset of cases. In many of these cases, genuine atypical features and dysplastic characteristics of the glands were noted at the interface of SH and AdCC. To investigate this phenomenon further, 88 sinonasal AdCC cases were selected from the authors’ files and analyzed histologically, immunohistochemically, and genetically searching for *MYB/MYBL1* and *NFIB* gene fusions. HPV testing was also performed. Univariate statistical analysis was conducted on our cohort. Thirty-one cases (35%) showed features of atypical sinonasal glands arising in SH (ASGSH) at the SH-AdCC interface, characterized by bilayered epithelium, architectural disarray, mild nuclear polymorphism, and atypia, sometimes with colloid-like material in the lumen. The MYB immunomarker was negative in 14 ASGSHs (with a positive internal control in AdCC cells), while only two cases showed faint and moderate to weak expression of the antibody in ASGSH glands. In 12 cases, the immunostaining of ASGSH could not be properly assessed, while AdCC cells were negative. The immunostaining was not performed in five cases. Our findings suggest that a subset of sinonasal AdCC may originate in a multistep dysplastic process within SH, consistent with an SH-ASGSH-AdCC progression sequence.

**Supplementary Information:**

The online version contains supplementary material available at 10.1007/s00428-025-04053-1.

## Introduction

Sinonasal AdCC is a rare salivary-type malignancy of the head and neck that presumably arises from minor salivary or seromucinous glands (1). These tumors exhibit a polymorphic histology, often combining tubular, cribriform, and solid growth patterns. However, other rare morphological patterns, such as trabecular, glandular, anastomosing cords, cystic spaces/macrocystic, epithelial-myoepithelial carcinoma-like, and pleomorphic adenoma-like patterns, may also be encountered. Furthermore, metatypical changes, including squamous cells, signet ring cells, tubular hypereosinophilia, and sebaceous cells, may also occur (1–7). Sinonasal AdCCs are in most cases defined by canonical *MYB/MYBL1::NFIB* gene fusions, although non-canonical fusions and fusion-negative cases have also been reported (4, 8–12).

Although it has been generally accepted that sinonasal AdCCs probably originate from seromucinous glands, their histogenesis and their putative precursor lesions, if any, have never been verified. The role of background mucosa or glands in the development of sinonasal AdCC, particularly the presence of atypical or dysplastic features, has received little attention in surgical pathology reports and the literature.

We have long been aware of an association between SH/ REAH and sinonasal AdCC (13, 14), often with a transitional interface area with presenting atypical sinonasal glands arising in SH (ASGSH) as described previously by our group (15). The conjecture that SH/REAH represents rather a neoplastic sinonasal lesion than a hamartoma was described in few studies (16–18). Molecular studies investigating genetic alterations in SH/REAH and malignant tumors associated with SH/REAH are limited. One study identified monoclonality in a case of SH using the HUMARA assay, indicating a clonal origin of these lesions. Additionally, an *EGFR::ZNF267* gene fusion was detected in one case of SH (19). Another case of low-grade non-intestinal type adenocarcinoma arising in association with REAH was found to harbor an *FN1::NRG1* gene fusion (20). Recently, atypical adenomatous lesions arising in SH/REAH were reported to harbor mutations in the *BRAF* gene (2/10 cases), *RET* gene (2/10 cases), and *FAT1* gene (1/10 case) (15).

Sinonasal AdCC are characterized by non-specific clinical symptoms often mimicking chronic rhinosinusitis. Despite their prolonged clinical course, which typically includes bone destruction, perineural involvement (21), and a low incidence of regional metastases (22), they frequently develop local recurrences and distant metastatic dissemination, mainly to the lungs, bones, and liver (23). Metastatic spread is common even with clear surgical margins several years or decades after initial diagnosis and treatment (1). Complete surgical removal of the tumor is the gold standard, often followed by radiotherapy for residual microscopic disease (24–26). However, the prognosis usually remains poor due to the anatomical location with vital structures in close proximity to the main tumor mass, which limits adequate surgical removal (1, 25, 27–29). Additionally, perineural spread may cause skip lesions beyond a clear surgical margin and be responsible for local recurrence. Thus, a clear margin is not always a reliable guideline (1).

To address the hypothesis that a subset of sinonasal AdCCs originate from SH/REAH via a transitional stage involving atypical sinonasal glands within SH (ASGSH). We collected a series of 88 sinonasal AdCC cases. Molecular genetic testing using the TruSight Oncology 500 Kit and fluorescence in situ hybridization (FISH) was performed to identify both canonical and non-canonical gene fusions as well as potentially targetable gene mutations. In four cases, microdissection was conducted to separately analyze SH and AdCC components.

## Materials and methods

### Case selection

The cases were retrospectively retrieved from the consultation files of the Tumor Registry at the Department of Pathology, Faculty of Medicine in Pilsen and Bioptic Laboratory, Ltd in Pilsen, Czech Republic, and tumor registries of the co-authors. A total of 100 cases were finally collected. Eleven HPV positive cases were excluded from the study, and the diagnosis of HPV-associated multiphenotypic carcinoma was established. One tumor was excluded based on additional clinical information about primary AdCC of submandibular gland followed by secondary involvement of sinonasal area. In total, a cohort of 88 cases of sinonasal AdCC was included in the current study and further examined.

The tumors were examined histologically, immunohistochemically, using next-generation sequencing (NGS) and/or fluorescence in situ hybridization (FISH) looking for *MYB/MYBL1* and/or *NFIB* gene fusions or any novel gene fusions/mutations. Four cases with available tissue blocks and distinct structures of SH and AdCC were macrodissected and tested separately. The HPV DNA detection was performed using a set of several PCRs with different primers **(**Table 1**)** to cover a wide detection range of predominantly high- and low-risk HPV types. Where available, clinical follow-up was obtained from the patients, their physicians, or referring pathologists.

**Table 1:** Primers of the human papilloma virus infection

HPV	Type 16	TCA AAA GCC ACT GTG TCC TGA
		CGT GTT CTT GAT GAT CTG CAA
	Type 18	CCG AGC ACG ACA GGA ACG ACT
		TCG TTT TCT TCC TCT GAG TCG CTT
	Type 31	CTA CAG TAA GCA TTG TGC TAT GC
		ACG TAA TGG AGA GGT TGC AAT AAC CC
	Type 33	AAC GCC ATG AGA GGA CAC AAG
		ACA CAT AAA CGA ACT GTG GTG
	Type 35	CCC GAG GCA ACT GAC CTA TA
		GGG GCA CAC TAT TCC AAA TG
	Type 45	TTA AGG ACA AAC GAA GAT TTC ACA
		ACA CAA CAG GTC AAC AGG ATC TAA
	CPSGB	ATA TGT CTG AGC CTC CWA ART T
		ATG TTA ATW SAG CCW CCA AAA TT
		TTA TCA WAT GCC CAY TGT ACC AT

Histologically, we focused on the presence or absence of REAH and SH and their architecture together with the presence of atypical features close to the malignant component.

This study was approved by the Ethics Committee of the Faculty Hospital in Pilsen and Charles University, Faculty of Medicine in Pilsen, Czech Republic, on August 2, 2018.

### Histology and immunohistochemistry

For conventional microscopy, the excised tissues were fixed in formalin, processed routinely, embedded in paraffin (FFPE), cut, and stained with hematoxylin and eosin.

For immunohistochemistry, 4-μm-thick sections were cut from paraffin blocks and mounted on positively charged slides (TOMO, Matsunami Glass IND, Osaka, Japan). Sections were processed on a BenchMark ULTRA (Ventana Medical Systems, Tucson, AZ), deparaffinized and subjected to heat-induced epitope retrieval by immersion in a CC1 solution (pH 8.6) at 95 °C. The following antibodies were used: AE1/3 (AE1/AE3 + PCK26, ready to use [RTU], Dako), CK7 (OV-TL 12/30, 1:200 dilution, Dako), CK14 (SP53, 1:800 dilution, Cell Marque), Ki-67 (MIB-1, RTU, Dako), MYB (EP769Y, 1:100 dilution, AbCam), p63 (DAK-p63, RTU, Dako), p40 (DAK-p40, RTU, Biocare Medical), p16 (R15-A, rabbit monoclonal antibody, 1:100 dilution, DB Biotech), SOX10 (SP267, RTU, Cell Marque), and S100 protein (polyclonal rabbit antibody, RTU, Dako).

Visualization of bound antibodies was performed using the ultraView Universal DAB Detection Kit (Roche, Tucson, AZ) and ultraView Universal Alkaline Phosphatase Red Detection Kit (Roche, Tucson, AZ). The slides were counterstained with Mayer’s hematoxylin. Appropriate positive and negative controls were employed.

In four cases, where sufficient non-tumor material was available, the samples were macrodissected into two parts separating the tumor and non-tumor components. DNA was extracted using the QIAsymphony DNA Mini.

### Molecular genetic study

#### TruSight Oncology 500 Kit (TS500)

Mutation analysis and fusion transcript detection were performed using TruSight Oncology 500 Kit (Illumina, San Diego, CA). RNA was extracted using the Maxwell RSC DNA FFPE Kit and the Maxwell RSC Instrument (Promega, Madison, WI) according to the manufacturer’s instructions and quantified using the Qubit HS RNA Assay Kit (Thermo Fisher Scientific, Waltham, MA). DNA was extracted using the QIAsymphony DSP DNA mini (Qiagen, Hilden, Germany) and quantified using the Qubit BR DNA Assay Kit (Thermo Fisher Scientific, Waltham, MA). The quality of DNA was assessed using the FFPE QC kit (Illumina), the quality of RNA using Agilent RNA ScreenTape Assay (Agilent, Santa Clara, CA). DNA samples with Cq < 5 and RNA samples with DV200 ≥ 20 were used for further analysis. After DNA enzymatic fragmentation with KAPAFrag Kit (KAPA Biosystems, Wilmington, MA), DNA and RNA libraries were prepared with the TruSight Oncology 500 Kit (Illumina) according to the manufacturer’s protocol. Sequencing was performed on the NovaSeq 6000 sequencer (Illumina) following manufacturer’s recommendations. Data analysis was performed using the TruSight Oncology 500 v2.2 Local App (Illumina). Variant annotation and filtering were performed using the Omnomics NGS analysis software (Euformatics, Espoo, Finland). Custom variant filter was set up including only non-synonymous variants with coding sequences and read depth greater than 50, while benign variants according to the ClinVar database (30) were excluded. The remaining subset of variants was checked visually, and suspected artifactual variants were excluded.

#### FISH analysis

Before performing FISH, hematoxylin and eosin-stained slides were examined to determine the areas for cell counting. Then, a 4-µm-thick formalin-fixed, paraffin-embedded section was placed onto a positively charged slide. The unstained slide was routinely deparaffinized and incubated in the 1 × Target Retrieval Solution Citrate pH 6 (Dako, Glostrup, Denmark) for 40 min at 95 °C, subsequently cooled for 20 min at room temperature in the same solution and washed in deionized water for 5 min. The slide was digested in protease solution with pepsin (0.5 mg/mL) (Sigma-Aldrich, St Louis, MO, USA) in 0.01 M HCl at 37 °C from 45 to 60 min according to the sample conditions. The slide was then rinsed in deionized water for 5 min, dehydrated in a series of ethanol solutions (70%, 85%, 96% for 2 min each), and air-dried.

The details of *EWSR1*, *MYB*, *NFIB* break-apart, and the *MYB*::*NFIB* fusion analysis have been described previously (31, 32)**.** For the detection of *EWSR1*::*MYB* fusion, custom designed EWSR1::MYB dual fusion probes comprising of catalogue 22q12.2. EWSR1 DF 498 kb probe and custom MYB probe with chromosomal location: chr6:135,271,382–135,771,382 (Agilent Technologies, Santa Clara, California, USA) were used following similar protocols.

#### Detection of HPV

For HPV studies, genomic DNA was isolated from formalin-fixed, paraffin-embedded tissue using QIAsymphony SP, and, moreover, special precautions were taken to prevent HPV DNA microcontamination. Briefly, five 5-μm-thick sections were cut from the blocks. A new microtome blade was used each time a new case was sectioned. DNA was extracted by the QIAsymphony DNA Mini Kit (Qiagen, Hilden, Germany) according to manufacturer’s protocol. The quality of isolated DNA was checked by PCR that amplificates set of control genes (33).

The HPV DNA detection was performed using a set of several PCRs with different primers **(**Table 1**)** to cover a wide detection range of predominantly high- and low-risk HPV types. For all samples, the primer’s systems targeting both L1 and E1 region were used: CPSGB, GP5 + /GP6 + , as previously described (34). To avoid false negative findings (because of loss of L1 or E1 region due a process of HPV integration into host genome), PCR targeting HPV oncogenes E6 and E7 of six most prevalent HR-HPV types, namely types 16, 18, 31, 33, 35, and 45, was performed (35).

All PCR were run on the cycler GeneAmp PCR System 9700 (PE/Applied Biosystem, Forster City, CA). Amplicons were analyzed in 2% agarose gel with ethidium bromide. Positive PCR samples were genotyped by hybridization to type-specific probes or sequenced and compared to BLAST databases. Positive and negative controls were included in every run.

#### Statistics description

Statistical analysis was performed using SW SAS (Cary, NC, USA). The descriptive statistics such as absolute and relative frequencies, mean, standard deviation, variance, median, interquartile range, minimum, and maximum were calculated. The nonparametric tests (Wilcoxon two-sample test) were used to determine the distribution differences of parameter solid between given therapies. The Kaplan–Meier analyses have been used to calculate the overall survival. The Gehan-Wilcoxon test, log-rank test, and Cox regression hazard model including hazard ratio calculations have been used to assess the clinical impact of examined covariates. Statistical significance was determined at the level of 5%.

## Results

### Demographic and clinical features

## Demographic and clinicopathological findings

The cohort included 88 cases of sinonasal AdCC previously reported by our group (article accepted, in press). Clinical data, follow-up information, and molecular genetic results are detailed in Table 2 and **Supplementary Table 1**.

**Table 2:** Clinicopathological characteristics of all patients with sinonasal AdCC (no = 88)

Gene alterations		RNA panel				FISH		DNA panel		
	Total (%)	*MYB::* *NFIB*	*MYBL1::* *NFIB*	Non-canonical fusions	Fusion/break Negative	*MYB, NFIB,* EWSR1 break*	NA/ND	Mutations Oncogenic	Gene mutations of UPS	NA/ND
Number of cases	88 (100%)	49 (57%)	9 (10%)	4 (4%)	6 (7%)	9 (10%)	11 (13%)	22/31 (71%)	24/31 (77%)	57
Age (y)										
< 65	51	30	5	2	4	5	5	16	16	33
More than 65	35	17	4	2	2	4	6	7	9	24
Unknown	2	2	0	0	0	0	0	0	0	2
Median	58.8									
Rrange	22–86									
Sex										
Male	45	26	4	4	2	3	6	12	12	29
Female	41	21	5	0	4	6	5	10	12	26
Unknown	2	2	0	0	0	0	0	0	0	2
Primary tumor site										
Nasal cavity	49	28	7	2	4	5	3	14	13	31
Maxillary sinus	26	14	1	1	1	3	6	4	7	17
Sphenoid sinus	8	5	1	1	0	1	0	3	3	5
Ethmoid sinus	4	2	0	0	1	0	1	1	1	3
Epipharynx	1	0	0	0	0	0	1	0	0	1
Follow-up available (mo)	60									
Range	1–276									
Mean	62.7									
Outcome	60									
Alive NED	17	11	2	1	3	0	0	7	7	7
Alive WD	10	8	1	0	0	0	1	5	4	4
DOD	18	10	1	2	0	2	3	4	4	12
DOC	4	2	0	0	0	2	0	0	0	4
Lost to FU	11	6	1	0	1	2	1	2	3	8
Not available	28	12	4	1	2	3	6	5	6	22
Metastases	14	9	2	0	0	2	1	4	3	9
Recurrences	26	16	1	2	3	2	2	12	10	12

* AdCC*, adenoid cystic carcinoma; *DOD*, dead of disease; *DOC*, dead of other causes; *FU*, follow-up; *NA*, not analyzable; *NED*, no evidence of disease; *ND*, not done because tissue was not available; *UPS*, uncertain pathogenetic significance; *WD*, alive with disease

^**^Fusion negative cases, but harboring *MYB* (no = 7), *NFIB* (no = 1), or *EWSR1* (no = 1) gene break

The patients were 45 men and 41 women (with gender unknown in two cases), ranging in age from 20 to 86 years (mean 58.8 years, median 60.5 years). Clinical data were unavailable for 31 patients. The tumors were located in the nasal cavity (*n* = 49), maxillary sinus (*n* = 26), sphenoid sinus (*n* = 8), ethmoid sinus (*n* = 4), and epipharynx (*n* = 1).

Treatment involved excision or radical surgical resection in 38 cases. Chemotherapy, radiation, and/or proton therapy were used either before or after surgery or as the sole therapeutic approach in 11, 36, and 5 patients, respectively. One patient received targeted therapy with imatinib. Metastatic spread occurred in 14/54 cases (26%), with metastases affecting the lung (*n* = 9), liver (*n* = 2), brain (*n* = 1), bones (pelvis and sacrum; *n* = 1), and axilla (*n* = 1). Recurrence was observed in 26/54 cases (47%) **(**Table 2**)**.

Follow-up status was available for 61/88 patients (70%), with the follow-up period ranging from shortly after diagnosis to 276 months (mean 62.7 months, median 12 months). Seventeen/61 patients (28%) were alive without evidence of disease (mean, 92.2 months), 10/61 patients (16%) were alive with disease (mean, 43.8 months), 19/61 patients (31%) died of the disease (mean, 63 months), 4/61 patients (7%) died of unrelated causes (mean, 46.3 months), and 11/61 patients (18%) were lost to follow-up after a known period of regular check-ups (mean, 33.6 months) (Table 2). In 27 cases (31%), the follow-up information was unavailable.

### Histological features

The relationship between the usual and unusual histological appearances, type of cells and metatypical features, and the distribution of fusions, breakpoints, and mutations is presented in Table 3. Among the 88 collected cases (both with canonical and non-canonical gene fusions) of sinonasal AdCC, the histological appearance was characterized by a combination of tubular, cribriform, and/or solid growth patterns. Tubular pattern was present in 59/88 cases (67%), cribriform pattern in 70/88 cases (80%), and solid growth in 66/88 cases (75%). In 11 cases, solid growth was the only pattern observed in the tumor.

**Table 3:** The relationship between usuall and unusuall histological appearances, type of cells and metatypical features, and the distribution of fusions, breakpoints, and mutations

Gene alterations		RNA panel				FISH		DNA panel		
	Total (%)	*MYB::* *NFIB*	*MYBL1::* *NFIB*	Non-canonical fusions	Fusion/break Negative	*MYB, NFIB,* EWSR1 break*	NA/ND	Mutations Oncogenic	Gene mutations of UPS	NA/ND
Number of cases	88 (100%)	49 (57%)	9 (10%)	4 (4%)	6 (7%)	9 (10%)	11 (13%)	22/31 (71%)	24/31 (77%)	57
Basic growth patterns										
Tubular/cribriform (non-solid)	22	10	3	0	1	2	6	3	2	18
Solid 100%	11	4	0	1^a^	4	1	1	5	4	6
Tubular/cribriform/solid	55	35	6	3^b,c,d^	1	6	4	14	18	23
Unusual patterns										
Trabecular	18	9	2	2^b,d^	3	0	2	4	5	13
Single cells/single cell lines	12	9	0	1^b^	0	1	1	2	3	9
Macrocystic/pulmonary edema-like	12	5	1	1^c^	1	0	4	3	5	7
Pseudoglandular	10	4	0	0	2	1	3	1	1	9
Nested	4	2	1	0	0	1	0	0	0	4
Pseudopapillary	4	2	0	0	0	2	0	1	1	3
Glomerular	3	2	0	0	1	0	0	3	2	0
Tissue culture-like	3	1	0	0	0	1	1	1	0	2
Microlumens and reticular	3	1	1	0	0	1	0	0	0	3
Cell type										
Clear cells	13	7	3	2^b,d^	0	0	1	5	5	8
Clear vacuolated cells/signet ring cells	6	4	1	1^c^	0	0	0	3	3	3
Small cells	10	7	0	0	1	1	1	2	2	8
Squamous cells	4	3	0	0	1	0	0	2	2	2
Sebaceous cells	2	2	0	0	0	0	0	1	1	1
Myoepithelial-like	2	0	1	1^c^	0	0	0	0	1	1
Tubular hypereosinophilia	14	8	1	1^d^	1	1	2	7	6	7
Association to SH/REAH/ASGSH										
AdCC only	40	23	3	2^c,d^	3	4	5	9	12	27
SH/REAH/ASGSH present	31	18	4	1^b^	1	1	6	8	7	22
Intact surface without SH/REAH/ASGSH	17	8	2	1^a^	2	4	0	5	5	11

* ACTN4::MYB*^*a*^*, ESRRG::DNM3*^*b*^*, ACTB::MYB*^*c*^*, EWSR1::MYB*^*d*^

*AdCC*, adenoid cystic carcinoma; *ASGSH*, atypical sinonasal glands arising in seromucinous hamartoma; *REAH*, respiratory epithelial adenomatoid hamartoma; *SH*, seromucinous hamartoma

**Fig. 1:**
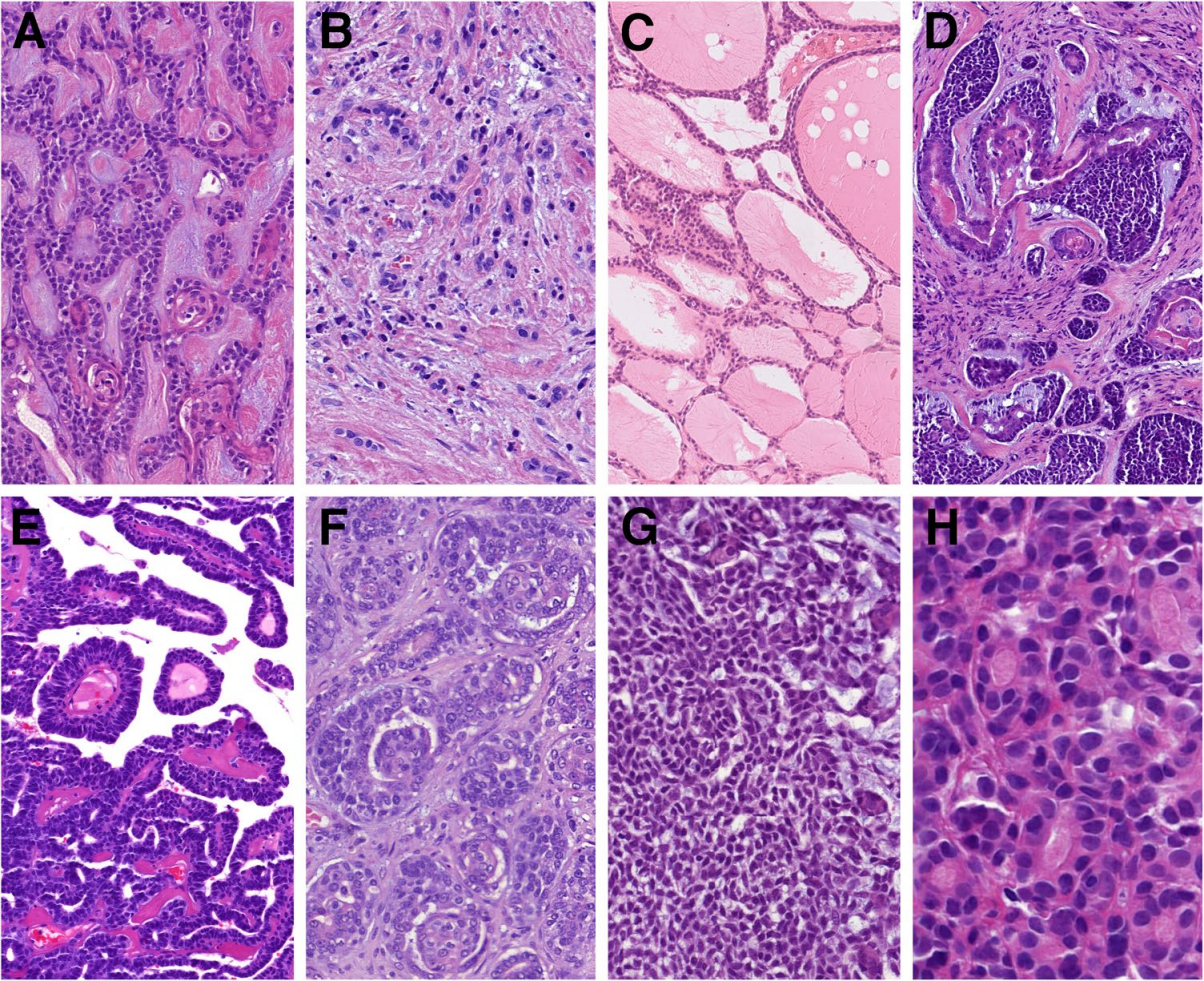
Less common growth patterns included trabecular (**A**), single cells/single cell lines (**B**), or macrocystic/pulmonary edema like (**C**). In ten cases, pseudoglandular architecture was present. In the depicted case, the glandular structures were peripherally composed of small cells with reduced, almost imperceptible cytoplasm, while the centrally located cells had eosinophilic cytoplasm, corresponding to tubular hypereosinophilia (**D**). Other rare patterns were pseudopapillary (**E**), glomeruloid (**F**), tissue culture-like (**G**), and two cases created microlumens (**H**)

Other less common growth patterns were trabecular (18/88, 20%) **(**Fig. 1A**)**, single cells or single cell lines (12/88, 14%) **(**Fig. 1B**)**, macrocystic/pulmonary edema like (12/88, 14%) **(**Fig. 1C**)**, pseudoglandular (10/88, 11%) **(**Fig. 1D**)**, nested (4/88, 5%), pseudopapillary (4/88, 5%) **(**Fig. 1E**)**, glomeruloid (3/88, 3%) **(**Fig. 1F**)**, tissue culture-like (3/88, 3%) **(**Fig. 1G**)**, microlumens (2/88, 2%) **(**Fig. 1H**)**, and reticular pattern (1/88, 1%).

**Fig. 2:**
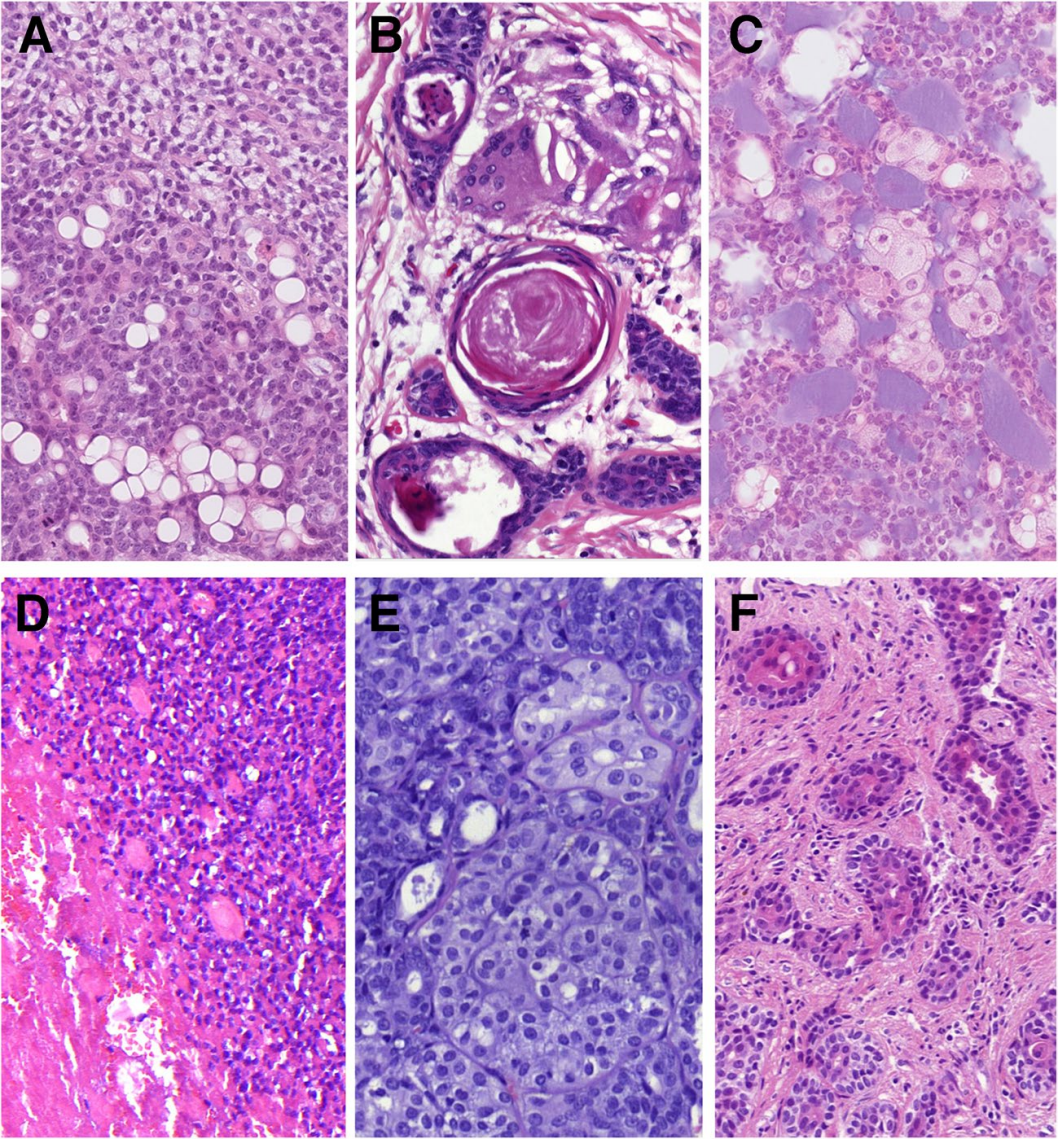
The spectrum of tumor cells varied, less common variants included clear cells with watery clear cytoplasm (*top*) and clear vacuolated cells/signet ring cells with excentric nuclei (*bottom*) (**A**). Squamous cells were present in four cases with keratin pearls and giant cell reaction in one case (**B**). Sebaceous cells were noted in two cases (**C**), and in one case were present large cells with eccentric, mildly eosinophilic cytoplasm forming tubules and small nests (**D**). Two cases exhibited features of myoepithelial carcinoma, in cases with a lobulated pattern, characterized by a hypercellular periphery and a hypocellular center (**E**). Tubular hypereosinophilia was present in 14 cases (**F**)

The tumors displayed a spectrum cellular morphologies, including conventional basaloid cells with varying nuclear appearances, ranging from low-grade to high-grade. Other rare variants observed included clear cells with watery clear cytoplasm (*n* = 13) **(**Fig. 2A**)**, small cells with minimal to absent cytoplasm (*n* = 10) **(**Fig. 1D**)**, clear vacuolated cells/signet ring cells with eccentric nuclei (*n* = 6) **(**Fig. 2A**)**, and squamous cells (*n* = 4) with keratin pearls in one case **(**Fig. 2B**)**. Additionally, sebaceous cells were noted in two cases **(**Fig. 2C**)**, while one case with a *MYB* gene rearrangement showed large cells with eccentric, mildly eosinophilic cytoplasm forming tubules and small nests **(**Fig. 2D**)**. Two cases exhibited features similar to myoepithelial carcinoma, with epithelioid or plasmacytoid cell morphology and eosinophilic cytoplasm. One of these cases also showed a lobulated pattern, characterized by a hypercellular periphery and a hypocellular center **(**Fig. 2E**)**. Tubular hypereosinophilia was present in 14 cases **(**Figs. 1D, 2F**)**.

Perineural spread and/or intraneural invasion was evident in 28 cases (31%), lymphovascular invasion in 18 cases (20%), bone invasion in 20 cases (23%), and muscle invasion was present in 9 cases (10%).

**Fig. 3:**
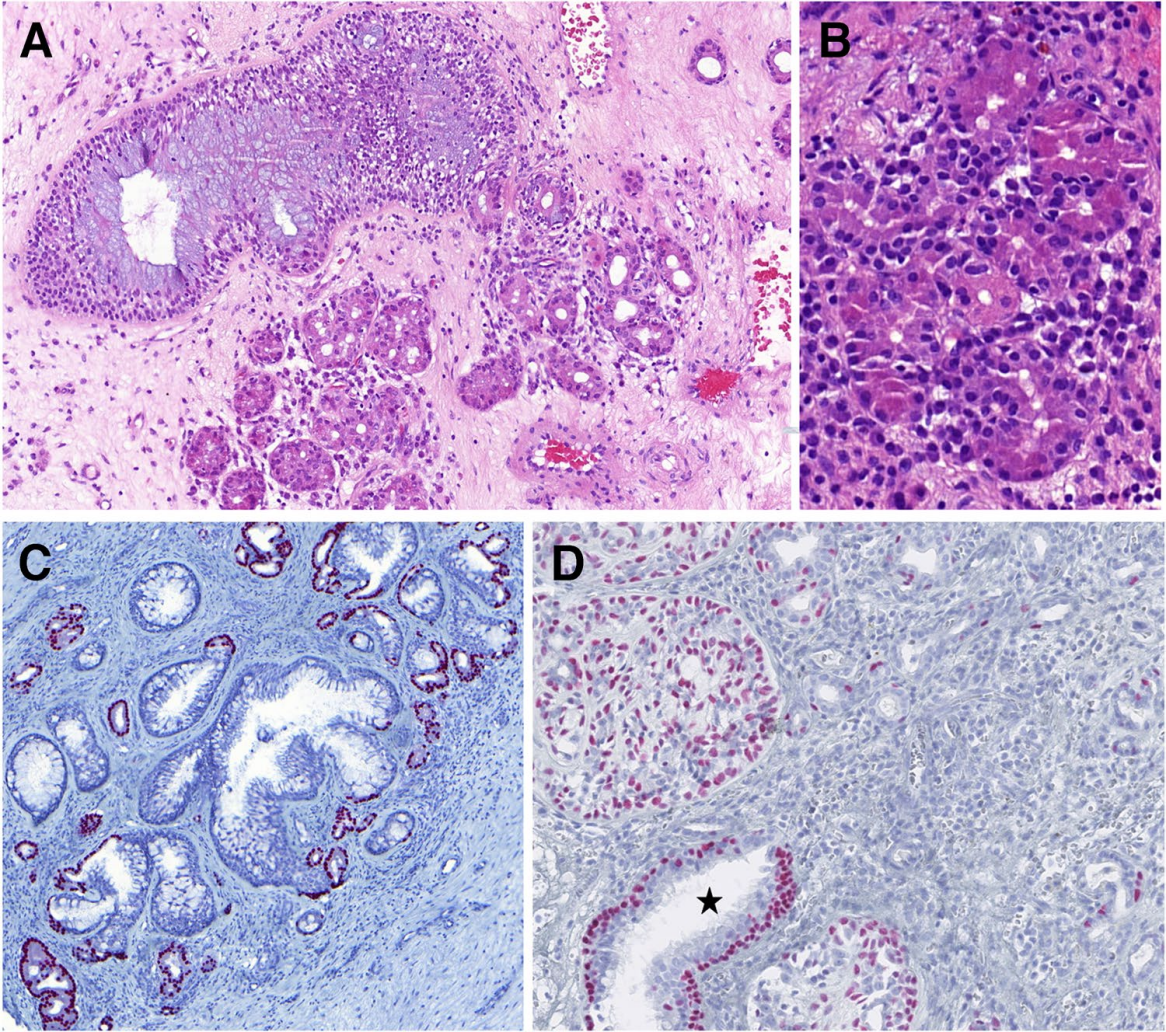
SH/REAH represent a continuum of lesions, with REAH glands showing sprouting of SH from their sides (**A**). SH usually retains a lobular architecture or is organized in small clusters, with the inner layer occasionally displaying coarse cytoplasmic granules (**B**). SH is positive for SOX10, while REAH is negative (**C**). p63/p40 is usually incompletely expressed in SH, reflecting its irregular and often missing myoepithelial layer, whereas it is consistently positive in myoepithelial portion of REAH (*star)* (**D**)

Forty cases in our cohort consisted of exclusively of tumor mass with no evidence of surface or other structures of normal mucosa. In 13 cases, normal sinonasal epithelium covered the tumor mass without evidence of atypia. In one case, the AdCC secondarily involved the respiratory epithelium. Additionally, in four cases (two from the nasal cavity and two from the maxillary sinus), squamous epithelium without dysplasia covered portions of tumor fragments. Overall, 31 cases (34%) were associated with SH, REAH, and/or atypical sinonasal glands arising in SH (ASGSH). The SH/REAH structures representing a continuum of lesions were present in at least a small focus. REAH glands had retained communication with the surface and showed characteristic sprouting of seromucinous glands into the stroma, indicative of an incipient stage of SH **(**Fig. 3A**)**. When present alone, SH retained its lobular architecture or was organized in small clusters primarily composed of a single cell, occasionally forming up to two layers **(**Fig. 3A**)**. The inner cell layer of SH was abundant, often coarsely eosinophilic and occasionally mucinous **(**Fig. 3B**)**. The outer cell layer was typically incomplete. Consistently, SH was positive for S100 protein and SOX10 **(**Fig. 3C**)** and showed no or only focal p63/p40 immunoexpression **(**Fig. 3D**)**, reflecting the incompleteness of the myoepithelial cell layer. Conversely, REAH displayed the opposite immunophenotype with S100/SOX10 negativity and consistent p63/p40 positivity in the intact myoepithelial cell layer.

**Fig. 4:**
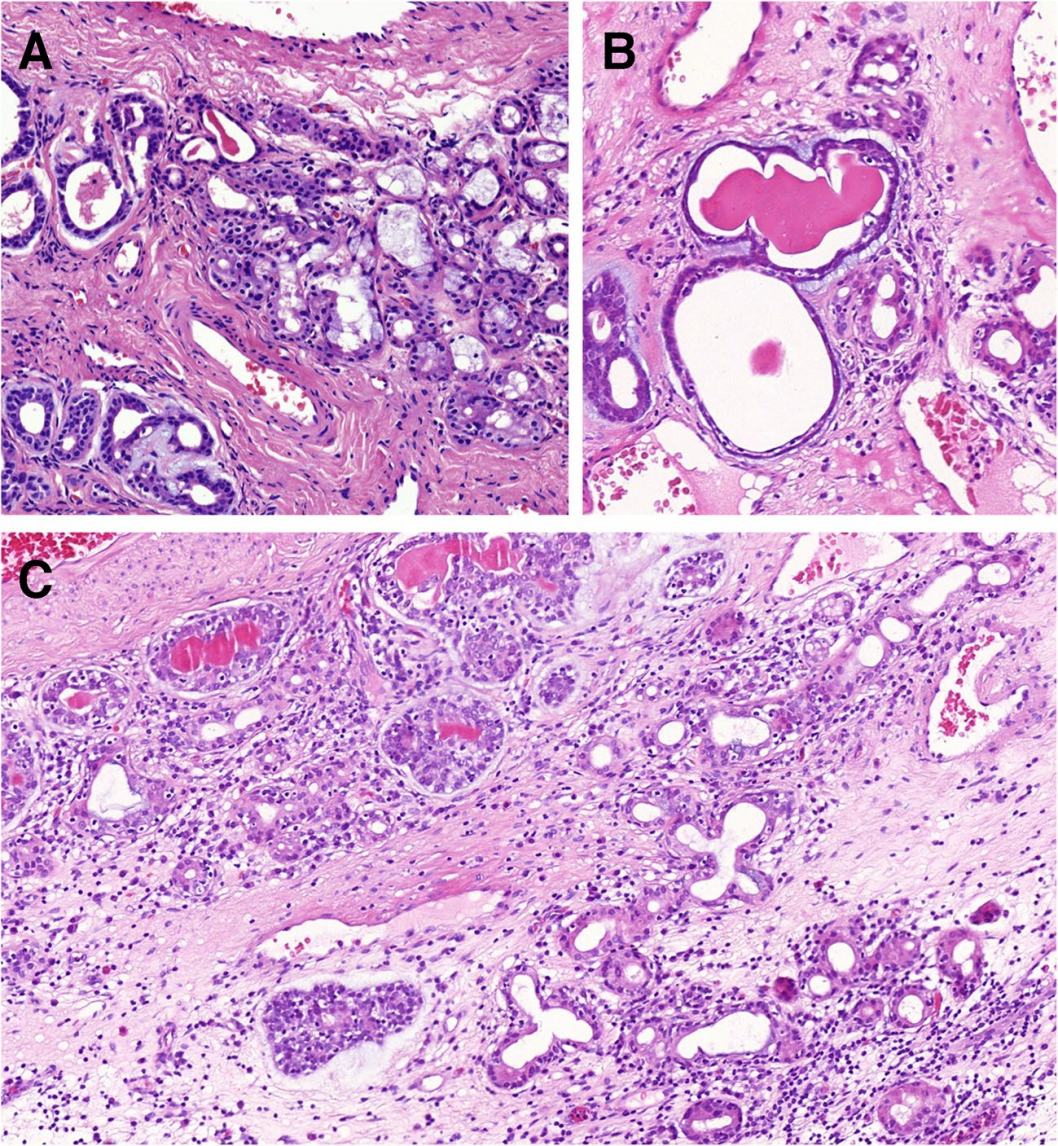
The ASGSH glands lost lobular arrangement typical for SH and were characterized by irregular and often discontinuous two-cell layers (**A**). ASGSH showed typical intraluminal cytoplasmic protrusions and contained material of different density in their lumens (**B**). The nuclei showed size and shape irregularities, were occasionally hyperchromatic, and in some cases, lost their polarity. ASGSH glands had a rounded or, more often, irregularly angulated and branched shape (*right*) (**C**)

**Fig. 5:**
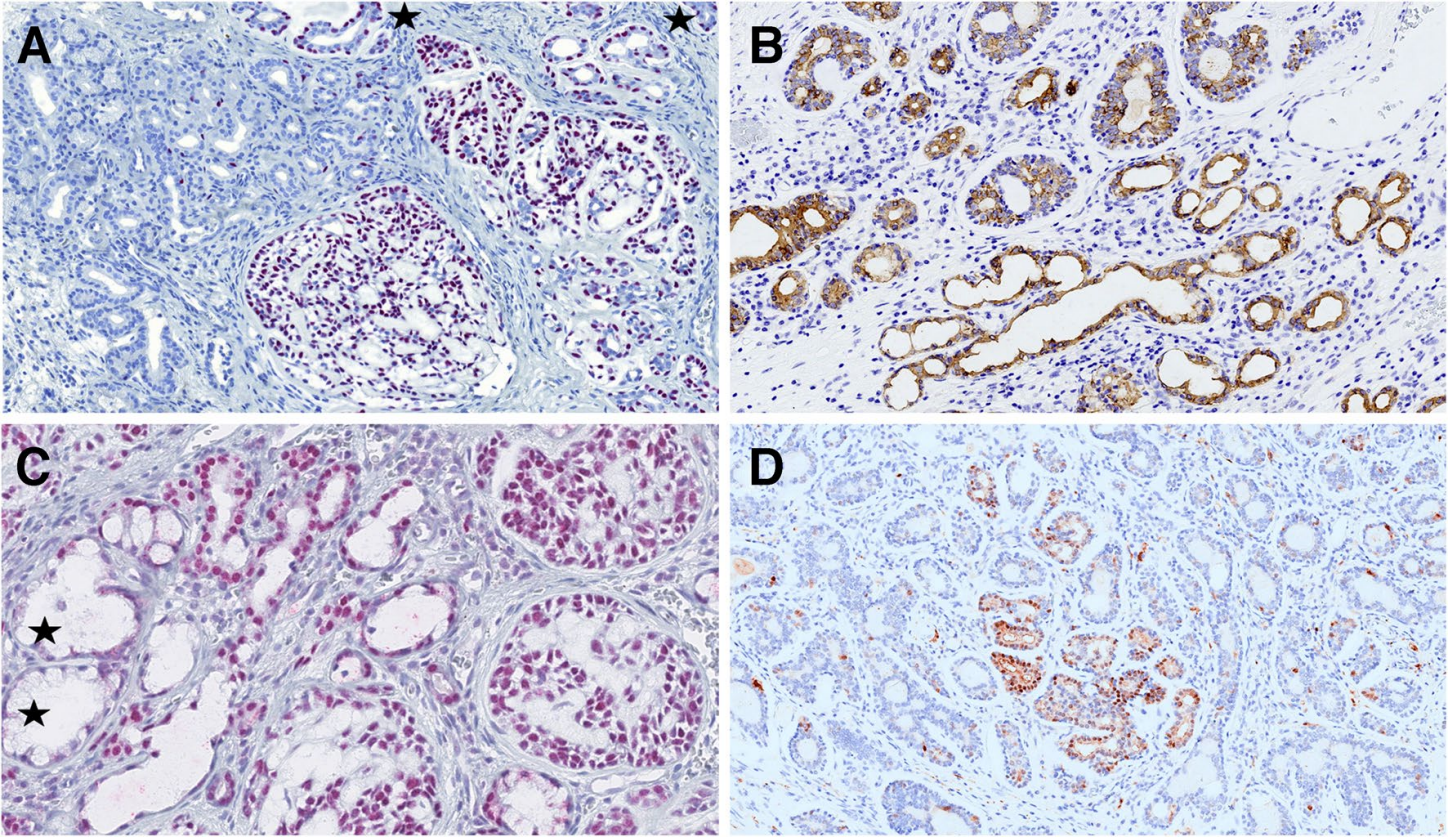
Abluminal cells of ASGSH were positive for p63, p40 (**A**), while luminal cells had reverse immunoexpression and were positive for CK7 (**B**). SOX10 was nuclear positive in AdCC (*right*) and SH/ASGHS (*center*) while negative in REAH (*left*) (**C**). The S100 protein effectively distinguished between SH and ASGSH glands, which were positive and dispersed as residual structures within the AdCC component, which was negative (**D**)

**Fig. 6:**
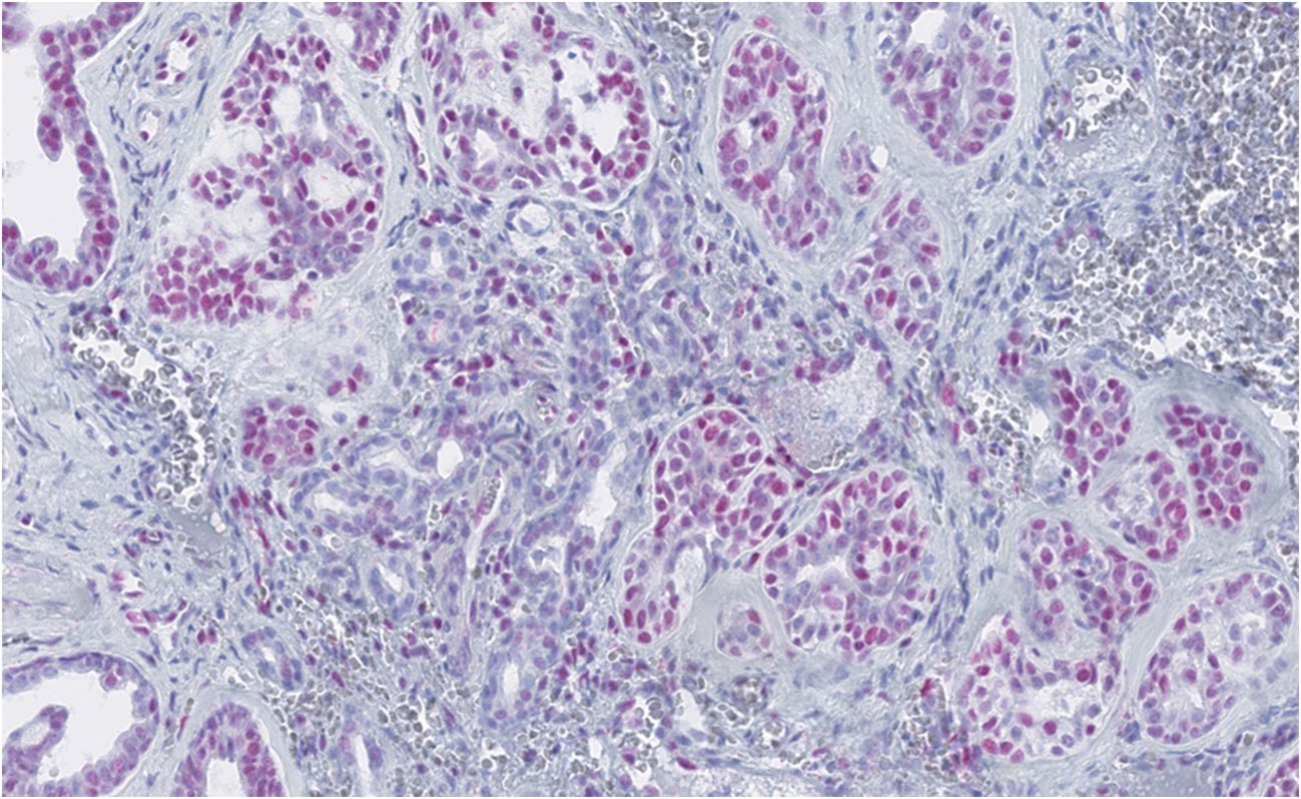
MYB expression was equivocal in two cases, the ASGSH component showed weak to moderate and faint nuclear expression, while AdCC showed strong nuclear pattern mostly in the abluminal layer

The ASGSH structures were devoted to SH and did not show direct communication with REAH structures. ASGSH structures were dispersed throughout the benign regions of SH/REAH and the genuine areas of AdCC. They lost the lobular arrangement typical for SH and were instead characterized by irregular and often discontinuous two cell layer pattern **(**Fig. 4A–C**)**. The inner layer frequently exhibited intraluminal cytoplasmic protrusions **(**Fig. 4B**)**. The nuclei showed size and shape irregularities and were occasionally hyperchromatic and in some cases lost their polarity. ASGSH glands had a rounded or, more often, irregularly angulated and branched morphology **(**Fig. 4B, C**)**. The lumen of ASGSH glands was often empty, but in some cases, varied amounts of material were present ranging from mildly eosinophilic secretion to dense, abundant magenta colloid-like material with peripheral clearing due to retraction **(**Fig. 4B**)**. Immunohistochemically, the myoepithelial cells of ASGSH were positive for p63 and p40 **(**Fig. 5A**),** while the luminal cells had a reverse immunoexpression and were positive for CK7 **(**Fig. 5B**)**. In some cases, distinguishing the ASGSH component from AdCC was challenging, as the two lesions were intermingled. SOX10 nuclear expression was observed in AdCC and SH while was absent in REAH **(**Fig. 5C**).** The S100 protein effectively differentiated SH/ASGSH glands and AdCC as the former were S-100 positive and appeared dispersed as residual structures within the S100-negative AdCC component **(**Fig. 5D**)**. The MYB immunomarker was negative in 12 cases (with a positive internal control in AdCC cells), and in 2 cases, the expression was equivocal, moderate to weak, and faint when compared to AdCC component expression **(**Fig. 6**)**. Immunohistochemical results could not be adequately assessed in 12 cases, partly due to decalcification in samples with bone invasion. In five cases, MYB staining was not performed. FISH analysis revealed *MYB* gene rearrangements in 17 cases. In two out of four cases with clearly distinct ASGSH and AdCC components, the *MYB* gene remained intact in ASGSH but was rearranged in AdCC.

Two typical examples of AdCC with ASGSH interface are available at https://pathpresenter.net/public/display?token=ae3bf416 and https://pathpresenter.net/public/display?token=30a50922.

**Fig. 7:**
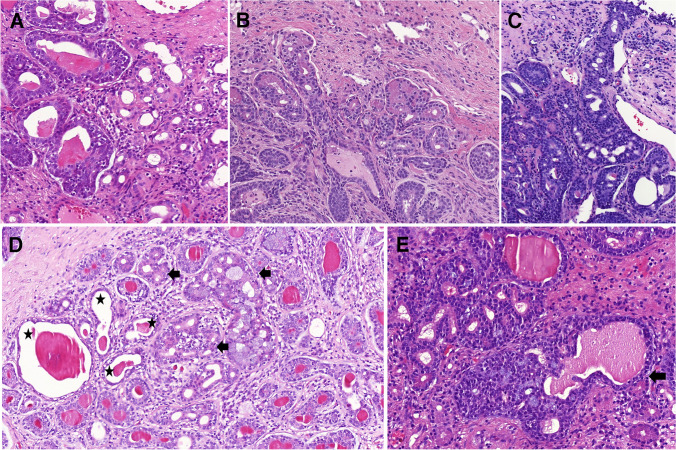
The ASGSH glands are characterized by irregular lumens with intraluminal cytoplasmic snouts and disarray of both secretory and outer myoepithelial layers and are connected to AdCC at the edge (**A**). An example of well-formed multilobular arrangements of structures with a histological appearance at the intersection of SH and ASGSH, where coarse eosinophilic granules are preserved at the periphery of the confluent structures, growing directly into AdCC (**B**). SH briefly descends from the respiratory surface and smoothly transitions to ASGSH, which exhibits a cribriform appearance and directly transforms into AdCC (**C**). Dispersed SH glands with a cribriform appearance (*arrows*), in part of mucinous nature and in part with coarse eosinophilic cytoplasmic granules, are admixed with irregularly shaped ASGSH glands with homogeneous eosinophilic luminal secretions (*star*) and embedded by tubular AdCC (*bottom left and right of the picture*) (**D**). A lobular arrangement of SH with eosinophilic granules in the cytoplasm (*left*), with a small group of glands (*arrow*), shows transition to dilated tubular structure of AdCC (**E**)

In those 31 cases, the ASGSH structures were either in the immediate vicinity **(**Fig. 7A**)** of the AdCC or there was a gradual transition or merging between the two lesions. In some cases, ASGSH glands were dispersed among the AdCC glands. A few cases in our cohort exhibited well-formed multilobular arrangement where coarse eosinophilic granules were preserved at the periphery of the confluent structures at the intersection of SH and ASGSH, growing directly into the AdCC **(**Fig. 7B**).** In some instances, residual SH displayed a cribriform architecture with a back-to-back pattern but with a well-preserved mucinous component, mostly forming a single cell layer **(**Fig. 7C–E). This pattern appeared to be somehow dripping down from the respiratory surface **(**Fig. 7C**)**.

### Molecular findings

Sinonasal AdCCs were predominantly characterized by canonical *MYB::NFIB* (49 cases) and *MYBL1::NFIB* (9 cases) fusions. In additional 9 cases of AdCC, rearrangements involving *MYB* (8 cases), *NFIB* (1 case), and *EWSR1* (1 case) were identified using FISH. Additionally, NGS revealed novel non-canonical fusion transcripts: *ACTBex3::MYBex3*; *ACTN4ex18::MYBex2*; *ESRRGex3::DNM3ex14*; and *EWSR1ex6::MYBex2*. Each of these was previously reported by our group (36). Genomic analysis identified mutations in genes with well-established roles in oncogenesis in 22/32 (69%) tumors. These included mutations in *NOTCH 1* and *NOTCH3* (4/22; 18%), *BCOR* (4/22; 18%), *EP300* (14/22; 9%), *KDM6A* (2/22; 9%), *SMARCA4* (2/22; 9%), *SPEN* (2/22; 9%), and *RUNX1* (2/22; 9%). Additional mutations were detected in *ARID1A*, *AVCR1B*, *CCND1*, *CHD2*, *CREBBP*, *DDX41*, *GEN1*, *LZTR1*, *MGA*, *MTOR*, *NF1*, *PALB2*, *PBRM1*, *PDGFRA*, *PHF6*, *PPM1D*, *PTEN*, *RB1*, *RIT1*, *ROS1*, and *TAF1*, each in one case (1/22; 5%). The molecular genetic data are detailed in our partner paper, which is currently in the second round of review. In one case, *MDM2* amplification was found by FISH and NGS. Additional 24 cases demonstrated a spectrum of gene mutations of uncertain pathogenetic significance. Notably, no well-known pathogenic or likely pathogenic variants were detected in 9/32 (28%) cases. In 54 cases, the tissue was not analyzable and in 3 cases not available for analysis.

### Statistical results

#### Overall survival (OS)

**Fig. 8:**
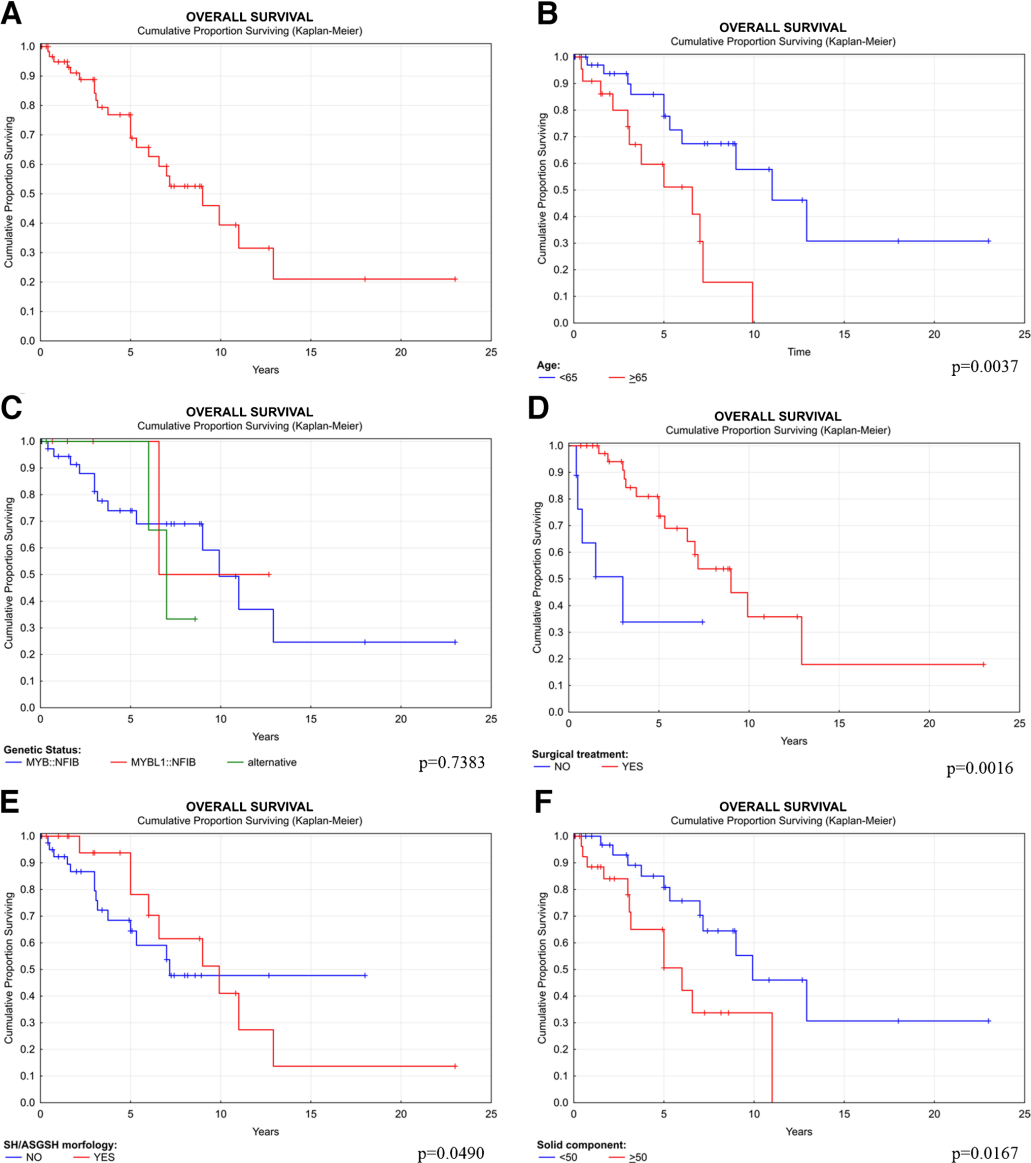
Overall survival of SC by Kaplan–Meier univariate analysis (**A**). Overall survival of sinonasal AdCC related to age (**B**), genetic status (**C**), surgical treatment (**D**), SH/ASGSH morphology (**E**), and the presence of solid component (**F**). Each graph with depicted *p*-value

Various clinical and histological parameters were separately investigated as potential predictors of patient outcome **(**Tables 4 and 5**)**. The 1-year, 5-year, and 10-year OS rates were found to be 95%, 69%, and 39%, respectively **(**Fig. 8A).

**Table 4:** Overall survival by selected clinical and histological parameters using the log-rank test and Wilcoxon test

Parameter	Subgroup (no.)	1-year OS (%)	5-year OS (%)	10-year OS (%)	Median (years)	Mean (SE) years
**Age**	< 65 (51)	97	78	58	6.0	9.42 (0.9035)
	≥ 65 (35)	90	60	15	3.0	5.35 (0.7912)
**Gender**	Female (41)	92	70	47	5.0	8.43 (1.0924)
	Male (45)	97	67	24	5.0	6.57 (0.5905)
**Gene fusion**	*MYB::NFIB* (49)	94	74	49	5.3	8.87 (0.9052)
	*MYBL1::NFIB* (9)	100	100	50	6.6	6.58 (NA)
	Non-canonical gene fusions (4)	100	100	33	6.0	6.67 (0.3849)
**Site**	Nasal cavity (49)	97	76	36	5.3	8.56 (0.9708)
	Maxillary sinus (26)	88	52	34	3.2	4.6 (0.5447)
	Sphenoid sinus (8)	100	80	53	3.0	8.33 (1.8086)
	Ethmoid sinus (4)	100	50	50	5.0	5.0 (NA)
	Epipharynx (1)	100	NA	NA	NA	NA (NA)
**Treatment**	Surgery (38)	100	74	36	5.3	8.53 (0.8323)
	No surgery (9)	63	34	NA	0.42	1.92 (0.4471)
	Chemotherapy (38)	81	50	NA	0.75	4.47 (0.8224)
	No chemotherapy (11)	97	73	38	5.3	8.55 (0.8923)
	Radiotherapy (36)	92	80	NA	5.0	6.03 (0.7626)
	No radiotherapy (13)	94	63	33	3.1	7.71 (0.8944)
**Metastasis**	Not present (40)	100	74	40	5.3	8.48 (0.8025)
	Present (14)	76	48	36	0.75	4.40 (0.7639)
**Recurrence**	Not present (29)	88	68	35	5.0	6.50 (0.7134)
	Present (26)	100	70	40	5.0	8.22 (0.9479)
**ASGSH**	Not present (57)	92	64	48	3.8	5.43 (0.4249)
	Present (31)	100	78	41	5.0	8.94 (1.0131)
**Solid component**	< 50% (55)	100	81	46	7.0	9.37 (0.8876)
	≥ 50% (33)	88	50	33	3.1	6.22 (0.9676)
**Metatypical pattern**	Not present (69)	93	72	45	5.3	8.41 (0.8286)
	Present (19)	100	60	24	2.2	6.81 (1.1319)
**LVI**	Not present (69)	100	72	40	5.3	8.43 (0.7801)
	Present (19)	80	60	45	3.2	4.79 (0.7140)
**PNI**	Not present (60)	92	67	26	5.0	7.63 (0.9099)
	Present (28)	100	73	55	3.8	8.17 (0.9539)
**Bone invasion**	Not present (68)	96	69	38	5.3	8.14 (0.7769)
	Present (20)	92	72	50	1.7	5.07 (0.5903)

*ASGSH*, atypical sinonasal glands arising in seromucinous hamartoma; *DSS*, disease-specific survival; *LVI*, lymphovascular invasion; *OS*, overall survival; *PNI*, perineural invasion; *SE*, standard error

**Table 5:** Overall survival by selected clinical and histological parameters with stated hazard ratios using the Cox regression hazard model

Clinical/histological factor	Hazard ratio	Confidence interval	*p*-value
Age ≥ 65 years	3.419	1.411–8.283	0.0065
Female gender	1.161	0.497–2.711	0.7300
*MYB::NFIB*	0.915	0.257–3.261	0.8908
*MYBL1::NFIB*	0.633	0.082–4.861	0.6601
Alternative gene fusion	1.683	0.366–7.747	0.5038
Maxillary sinus	1.627	0.657–4.026	0.2927
Nasal cavity	0.745	0.326–1.701	0.4843
Sphenoid sinus	0.943	0.276–3.223	0.9257
No surgery	4.811	1.639–14.122	0.0042
No chemotherapy	2.331	0.855–6.356	0.0981
No radiotherapy	1.472	0.421–5.139	0.5447
Metastasis	1.641	0.672–4.009	0.2768
Recurrence	0.967	0.421–2.223	0.9370
ASGSH	1.239	0.522–2.939	0.6269
Solid component ≥ 50%	2.732	1.156–6.457	0.0220
Metatypical pattern	1.338	0.518–.458	0.5477
Lymphovascular invasion	1.327	0.520–3.387	0.5542
Perineural invasion	0.610	0.247–1.503	0.2823
Bone invasion	0.904	0.305–2.677	0.0335

Age was the most significant predictor of OS, with a statistically significant cutoff at 65 years (*p* = 0.0037). Patients younger than 65 years had OS rates of 90%, 60%, and 15% at the same time points. The hazard ratio (HR) for disease progression in the older group was 3.419 (confidence interval [CI] = 1.411–8.283) **(**Fig. 8**).**

Gender did not appear to influence clinical outcome (*p* = 0.7276). The 5-year and 10-year OS rates were 70% and 47% for females and 67% and 24% for males, respectively. For women the HR was 1.161 (CI = 0.497–2.711).

The molecular genetic background was not a significant predictor of a worse clinical outcomes (*p* = 0.7383). Only fusion-positive cases were included in the analysis, while negative, non-analyzable, and gene-break-carrying tumors were excluded. Patients with *MYB::NFIB*, *MYBL1::NFB* and non-canonical fusion-positive AdCCs demonstrated similar 5-year and 10-year OS rates. Tumors carrying *MYB::NFIB* gene fusion had an HR of 0.915 (CI = 0.257–3.261), *MYBL1::NFIB* had an HR of 0.633 (CI = 0.082–4.861), and non-canonical gene fusions had an HR of 1.683 (CI = 0.366–7.747) **(**Fig. 8C**)**.

Tumor location did not significantly influence OS (*p* = 0.7611). Median survival was lowest in patients with tumors in maxillary and sphenoid sinuses, at 3.2 and 3.0 years, respectively. The HR was low for all locations: nasal cavity 0.745 (CI = 0.326–1.701), maxillary sinus 1.627 (CI = 0.657–4.026), and sphenoid sinus 0.943 (CI = 0.276–3.223).

Patients treated with surgery showed significantly better OS than the patients without surgery (*p* = 0.0016). The HR for patients without surgery was 4.811 (CI = 1.639–14.122) **(**Fig. 8D**).** Chemotherapy, however, did not show a statistically significant effect on OS compared to patients who did not receive chemotherapy (*p* = 0.0871). The HR for disease progression in patients without chemotherapy was 2.331 (CI = 0.855–6.356). Similarly, no significant difference in OS was observed between patients treated with and without radiotherapy (*p* = 0.5400). The HR for disease progression in patients not receiving radiotherapy was 1.472 (CI = 0.421–5.139).

The presence of metastasis did not significantly influence OS (*p* = 0.2690); however, the analysis was limited by data availability in 54 cases (in 34 cases by invalid time, censoring, or strata values). In 5-year survival, patients with metastatic spread had a worse outcome than those without metastatic spread, with survival rates of 48% and 74%, respectively. The presence of metastases was associated with an HR of 1.641 (CI = 0.672–4.009). Recurrence, similar to metastases, had no proven effect on OS (*p* = 0.9367). However, data were censored or deleted due to missing events. The HR for patients experiencing recurrence was 0.967 (CI = 0.421–2.223).

#### Histological results

The presence of the ASGSH component was not related to worse or better outcomes (*p* = 0.6243). The HR for patients with ASGSH was 1.239 (CI = 0.522–2.939) **(**Fig. 8E). In contrast, the solid tumor component was negatively associated with OS (*p* = 0.0490). The most statistically significant cut-off for the amount of solid component identified as 50% (*p* = 0.0167). Patients with tumors with less than 50% solid component had a 5-year OS rate of 81%, whereas patients with tumors with 50% or more solid component showed a reduced 5-year OS rate of 50%. The presence of more than 50% of solid component was associated with an HR of 2.732 (CI = 1.156–6.457) **(**Fig. 8F**).** We also tested the literature-based cut-off of 30% for the solid component but could not demonstrate a difference compared to our 50% cut-off (*p* = 0.0167 vs. *p* = 0.0575, respectively). The metatypical pattern did not show any statistically significant correlation with OS (*p* = 0.5443), and the HR was 1.33 (CI = 0.518–3.458).

Lymphovascular invasion, perineural invasion, and bone invasion showed no statistically significant influence on OS, with *p*-values of 0.5510, 0.2748, and 0.8538, respectively. The HRs for these variables, in the above-stated order, were 1.327 (CI = 0.520–3.387), 0.610 (CI = 0.247–1.503), and 0.904 (CI = 0.305–2.677), respectively.

#### Disease-specific survival (DSS)

**Fig. 9:**
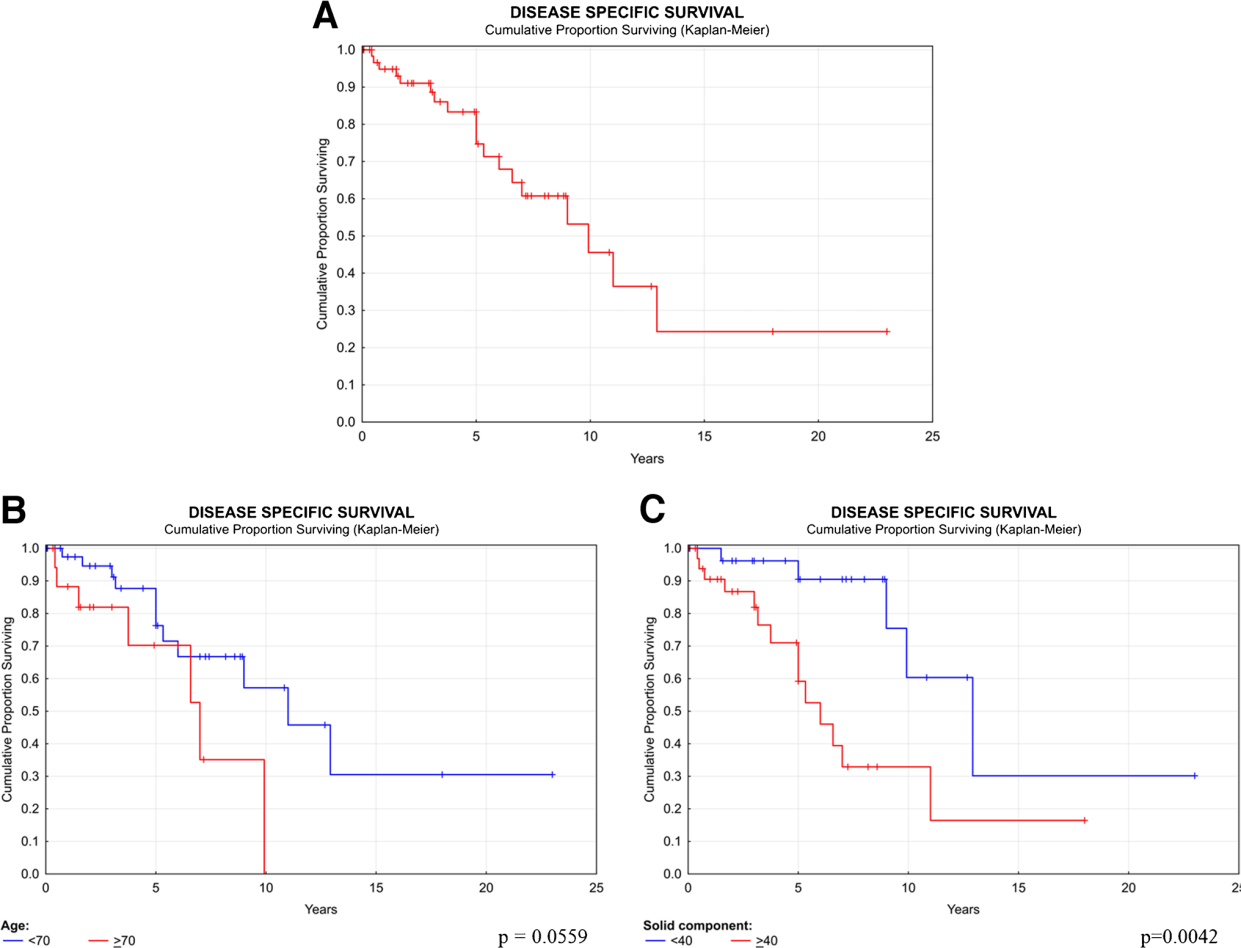
Disease-specific survival of SC by Kaplan–Meier univariate analysis (**A**). Disease-specific survival AdCC related to age (**B**) and the presence of solid component (**C**). Each graph with depicted *p*-value

In contrary to OS where any death is considered a negative event, disease-specific survival (DSS) only considers death directly attributable to the disease as a negative event. The DSS results were largely consistent with the OS data, with a few notable differences. The 1-year, 5-year, and 10-year DSS rates were calculated to be 95%, 75%, and 46%, respectively **(**Fig. 9A**)**. The median survival was 9.9 years. For DSS, the age cut-off was 70 years (*p* = 0.0559), with a median survival of 6 years for patients younger than 70 years and 3 years for those older than 70 years **(**Fig. 9B**)**. The presence of at least 40% solid component was significantly associated with worse DSS (*p* = 0.0042). The 5-year and 10-year survival rates for patients with less than 40% solid component were 91% and 59%, respectively, compared to 60% and 33% for those with more than 40% solid component **(**Fig. 9C**)**. The HR for > 40% of solid component was 4.033 (CI = 1.435–11.333). For detailed DSS data, please see the **Supplementary Tables 1 and 2.**

## Discussion

The sinonasal area is the second most common head and neck site for AdCC, a rare and aggressive malignancy (37). This cancer more frequently affects minor salivary glands compared to major salivary glands, with a prevalence of 60% versus 40%, respectively. The sinonasal tract represents some 25% of cases (37–39). Sinonasal AdCC manifests as a prolonged disease, often diagnosed at advanced stages in the TNM classification, with T3 and T4 stages representing 86% of cases (25, 37). Tumors originating in the sphenoid and maxillary sinuses are associated with the poorest prognosis due to their proximity to the skull base (40). Although our statistical analysis supported this observation, the significance of tumor location was low (*p* = 0.7611). The presence of a solid component is included in various grading systems and is recognized as an adverse prognostic factor (41–44). Some studies suggest that even a small proportion of solid growth negatively impacts prognosis (44), while others associate a threshold of more than 30% solid growth with worse outcomes (41). However, these studies primarily focus on salivary gland AdCC. In our analysis of sinonasal AdCC, solid growth in ≥ 50% of the tumor was associated with poorer OS, while solid growth in ≥ 40% correlated with poorer DSS.

Sinonasal AdCC belongs to the spectrum of salivary gland-type adenocarcinomas, arising from the seromucinous glands underlying the respiratory epithelium and possibly also from the surface epithelium as previously described (1, 45). We were long aware of an association of sinonasal AdCC with another concomitant tumor. SH and REAH are epithelial-glandular tumors representing a spectrum of lesions affecting upper respiratory tract that have been long considered non-neoplastic (2). Both are categorized as hamartomas, but the past investigations pointed towards neoplastic nature rather than an indolent non-neoplastic lesion (16–19). Ozolek et al. identified allelic imbalances in a subset of REAH, showing a mean fractional allelic loss of 31%, which was considered suspiciously high for a non-neoplastic lesion (16). The presence of allelic loss is common in many benign tumors including thyroid follicular adenoma, parathyroid adenomas (46, 47), or hemangiomas of the head and neck (48). The authors also demonstrated mutational similarities between REAH and sinonasal adenocarcinomas (SNAC), with both exhibiting the highest loss of heterozygosity on chromosomes 9p (CDKN2/p16) and 18q (DCC/DPC4).

Jo et al. performed histological and immunohistochemical analysis on a series of SNACs, among which six tumors showed a clear association to REAH (17). Another group examined the mitochondrial DNA mutation rate in SH, finding it significantly higher compared to normal seromucinous glands (18). Rooper et al. investigated a series of genetically heterogeneous low-grade non-intestinal type adenocarcinomas (non-ITAC). Among these, one case with an *FN1::NRG1* gene fusion was associated with REAH, characterized by prominent invaginations of the surface epithelium and basement membrane deposits within the REAH glands, as depicted in Fig. 1C, with overgrowth into adenocarcinoma (20). In our previous study, we performed immunohistochemical and genetic analyses of both REAH/SH and low-grade non-ITACs. Our findings revealed an immunohistochemical overlap between these entities. Molecular genetic analysis revealed an *EGFR::ZNF267* gene fusion in one SH, and monoclonality in another SH was confirmed using the HUMARA assay (19).

Our group previously suggested that SH/REAH and a transient lesion arising from SH, the ASGSH, are potentially neoplastic (15). ASGSH are typically irregular and have bilayered glands with an inner secretory layer and an outer, sometimes incomplete myoepithelial layer. The latter can occasionally be abundant, forming peripheral balls of myoepithelial cells when cut tangentially. The lumens often contain dense, eosinophilic, colloid-like material with artefactual separation and peripheral clearing. Moreover, we identified identical mutations in the *BRAF* (Val600Glu) gene in two cases and, in another two cases, a *RET* (Arg912Trp) gene mutation. In one case, an alteration in the *FAT1* (Pro1665Leu) gene was found. Recent studies have suggested that SH/REAH and ASGSH may participate in a multistep dysplastic process leading to the development of sinonasal malignancies, including low-grade tubulopapillary adenocarcinomas (19), recently described subset of sinonasal adenosquamous carcinoma (49) and the AdCC depicted in this study.

ASGSH is rarely reported in the literature and is often misinterpreted as altered non-tumorous content. Kwok et al. reported four cases of adenofibromatous solitary fibrous tumor with interspersed angulated glands containing intraluminal colloid-like material, identical to that observed in ASGSHs (50). It featured intraluminal cytoplasmatic snouting, disarray of both epithelial cell layers, and variations of nuclear size and shape, as well as colloid-like material in the lumen. In our registry, we identified similar cases with ASGSH glands surrounded by stroma with solitary fibrous tumor overgrowth, as seen in the whole slide image at https://my.pathomation.com/share/slide/VGJ76vaN7BGc2o0v9V72.

According to our analysis, the presence of the ASGSH component does not significantly affect clinical outcomes (*p* = 0.6243).

Similar to salivary gland AdCC, sinonasal AdCC is most commonly characterized by the presence of canonical *MYB/MYBL1::NFIB* gene fusion. Advanced methods of molecular pathology have identified additional non-canonical gene fusions involving *MYB* and *MYBL1*. These include *ACTB*, *ACTN4*, *C8orf34*, *EFR3A*, *EWSR1*, *FUS*, *PDCD1LG2*, *RAD51B* and *TGFBR3* as *MYB* partners, and *EWSR1* and *RAD51B* as *MYBL1* partner (4, 8–11). The *NFIB* gene has been involved in several fusions, including *NFIB::EPB41L2*, *MAP7::NFIB*, *NFIB::MCMDC2*, *C8orf34::NFIB*, *NFIB::CASC20*, and *NFIB::AIG1*, across salivary glands, sinonasal area, and non-head and neck locations (8, 11). Other non-*MYB/MYBL1::NFIB* gene fusions, such as *ESRRG::DNM3* and *TVP23C::CDRT4*, have also been reported in the literature and by our group (12, 36).

Additionally, studies have revealed various genetic mutations and pathway alterations in AdCC, including disruption in chromatin remodeling, DNA damage response, *NOTCH* signaling, and tyrosine kinase signaling (23, 51, 52). Further, disruptions in the *PI3K/Akt/mTOR* and *FGF/IGF/PI3K* pathways and other signaling cascades have been noted in AdCC (52, 53). These findings suggest that there are multiple genetic mechanisms contributing to the pathogenesis of AdCC that emphasize a complex genetic landscape of this tumor and point to potential targets for therapy beyond the canonical *MYB/MYBL1::NFIB* fusion (10).

In conclusion, we have described a series of 88 sinonasal AdCCs 31 of which were associated with SH/REAH and ASGSH. Our findings suggest that a subset of sinonasal AdCC may originate in a multistep dysplastic process within SH and an intermediate step of ASGSH. Moreover, unusual histological features of sinonasal AdCC were evaluated, and a statistical analysis of tumor outcome was performed.

### Study limitations

This is a retrospective study of 88 AdCC cases. However, follow-up was not available in 28 cases. Data on metastatic spread and recurrence were not available in 34 and 33 cases, respectively, due to invalid time, censoring, or strata values. For purposes of statistical testing, we revised the number of cases and performed the calculations using the actual numbers of available cases for each variable. The mean survival time and its standard error were underestimated because the largest observation was censored, and the estimation was restricted to the largest event time. Patients not available/lost to follow-up were assigned a survival time of 10 days from the start of the therapy/surgery. Thus, these patients were included in the analysis, but they did not significantly influence the results (the follow-up was only 10 days, after which they were lost from the analysis). Despite limitations, this study has analyzed one of the largest patient cohorts to date and provided insights into the clinical behavior of sinonasal AdCC.

## Supplementary Information

Below is the link to the electronic supplementary material.

Supplementary file1 (DOCX 22 KB)

Supplementary file2 (DOCX 17 KB)

## Data Availability

All data generated or analyzed during this study are included in this published article (and its supplementary information files). The preliminary results of the study were presented as a poster at the 113th Annual Meeting of the American and Canadian Academy of Pathology, held from March 23–28, 2024, in Baltimore, MD, USA.
