# Supplementary material for: Sinonasal adenoid cystic carcinomas accompanied by seromucinous hamartoma and/or atypical sinonasal glands arising from seromucinous hamartoma: insight into their histogenesis
Source: Virchows Arch. 2025 Feb 22;486(6):1269–87. doi: 10.1007/s00428-025-04053-1 (PMC12213922; doi:10.1007/s00428-025-04053-1)
Supplement: Supplementary file 1 — Supplementary file1 (DOCX 22 KB) [file 428_2025_4053_MOESM1_ESM.docx]

**Supplementary table 1.** Disease specific survival by selected clinical and histological parameters using the Log-rank test and Wilcoxon test.

| **Parameter** | **Subgroup (no.)** | **1-year OS (%)** | **5-year OS (%)** | **10-year OS (%)** | **Median (years)** | **Mean (SE) years** |
| --- | --- | --- | --- | --- | --- | --- |
| **Age** | <70 (62) | 97 | 76 | 57 | 6.0 | 9.42 (0.8534) |
|  | ≥ 70 (24) | 88 | 70 | NA | 3.8 | 6.46 (1.0678) |
| **Gender** | Female (41) | 92 | 70 | 53 | 5.0 | 8.77 (1.1170) |
|  | Male (45) | 97 | 77 | 27 | 6.0 | 7.18 (0.5544) |
| **Gene fusion** | *MYB::NFIB* (49) | 94 | 80 | 53 | 9.0 | 9.40 (0.8961) |
|  | *MYBL1::NFIB* (9) | 100 | 100 | 50 | 6.6 | 6.58 (NA) |
|  | Non-canonical gene fusions (4) | 100 | 100 | NA |  | 6.67 (0.3849) |
| **Site** | Nasal cavity (49) | 97 | 88 | 46 | 9.0 | 9.74 (0.9869) |
|  | Maxillary sinus (26) | 88 | 52 | 34 | 3.2 | 4.60 (0.5447) |
|  | Sphenoid sinus (8) | 100 | 80 | 53 | 3.0 | 8.33 (1.8086) |
|  | Ethmoid sinus (4) | 100 | 50 | NA | 5.0 | 5.0 (NA) |
|  | Epipharynx (1) | 100 | NA | NA | NA | NA (NA) |
| **Treatment** | Surgery (38) | 100 | 82 | 44 | 6.6 | 9.44 (0.8563) |
|  | No surgery (9) | 63 | 34 | NA | 0.42 | 1.92 (0.4471) |
|  | Chemotherapy (38) | 82 | 50 | NA | 0.75 | 4.47 (0.8224) |
|  | No chemotherapy (11) | 97 | 82 | 47 | 7.0 | 9.55 (0.9126) |
|  | Radiotherapy (36) | 94 | 68 | 39 | 5.0 | 8.41 (0.9303) |
|  | No radiotherapy (13) | 92 | 92 | NA | 7.0 | 6.48 (0.7052) |
| **Metastasis** | Not present (40) | 100 | 83 | 49 | 7.0 | 9.39 (0.8082) |
|  | Present (14) | 76 | 48 | 36 | 0.75 | 4.40 (0.7639) |
| **Recurrence** | Not present (29) | 88 | 72 | 43 | 5.0 | 6.94 (0.7110) |
|  | Present (26) | 100 | 75 | 45 | 5.3 | 8.81 (0.9564) |
| **ASGSH** | Not present (57) | 92 | 71 | 59 | 5.3 | 5.58 (0.4046) |
|  | Present (31) | 100 | 83 | 44 | 5.0 | 9.39 (0.9684) |
| **Solid component** | <40% (47) | 100 | 91 | 60 | 9.0 | 10.99 (0.9008) |
|  | ≥40% (41) | 91 | 59 | 33 | 3.8 | 6.48 (0.8290) |
| **Metatypical pattern** | Not present (69) | 93 | 77 | 53 | 6.0 | 9.09 (0.8430) |
|  | Present (19) | 100 | 67 | 27 | 3.0 | 7.33 (1.1306) |
| **LVI** | Not present (69) | 100 | 80 | 47 | 6.0 | 9.29 (0.7846) |
|  | Present (19) | 81 | 61 | 45 | 3.2 | 4.79 (0.7140) |
| **PNI** | Not present (60) | 92 | 73 | 31 | 5.3 | 8.37 (0.9560) |
|  | Present (28) | 100 | 78 | 58 | 5.0 | 8.54 (0.9509) |
| **Bone invasion** | Not present (68) | 92 | 76 | 45 | 6.6 | 8.91 (0.7950) |
|  | Present (20) | 92 | 70 | 52 | 1.7 | 5.07 (0.5903) |

ASGSH – atypical sinonasal glands arising in seromucinous hamartoma; DSS – disease specific survival; LVI – lymphovascular invasion; OS – overall survival; PNI – perineural invasion; SE – standard error
