# Supplementary material for: Sinonasal adenoid cystic carcinomas accompanied by seromucinous hamartoma and/or atypical sinonasal glands arising from seromucinous hamartoma: insight into their histogenesis
Source: Virchows Arch. 2025 Feb 22;486(6):1269–87. doi: 10.1007/s00428-025-04053-1 (PMC12213922; doi:10.1007/s00428-025-04053-1)
Supplement: Supplementary file 2 — Supplementary file2 (DOCX 17 KB) [file 428_2025_4053_MOESM2_ESM.docx]

**Supplementary table 2**. Disease-specific survival by selected clinical and histological parameters with stated hazard ratios using the Cox regression hazard model.

| **Clinical/histological factor** | **Hazard ratio** | **Confidence interval** | **p-value** |
| --- | --- | --- | --- |
| Age ≥ 70 years | 2.500 | 0.941-6.639 | 0.0659 |
| Female gender | 0.985 | 0.387-2.507 | 0.9745 |
| *MYB::NFIB* | 0.772 | 0.211-2.826 | 0.1526 |
| *MYBL1::NFIB* | 0.711 | 0.091-5.531 | 0.7446 |
| Alternative gene fusion | 2.055 | 0.432-9.786 | 0.8189 |
| Maxillary sinus | 2.132 | 0.824-5.520 | 0.1187 |
| Nasal cavity | 0.503 | 0.201-1.260 | 0.1426 |
| Sphenoid sinus | 1.143 | 0.326-4.001 | 0.8347 |
| No surgery | 7.213 | 2.254-23.083 | 0.0009 |
| No chemotherapy | 3.500 | 1.171-10.458 | 0.0249 |
| No radiotherapy | 1.748 | 0.386-7.910 | 0.4685 |
| Metastasis | 2.204 | 0.862-5.634 | 0.0990 |
| Recurrence | 1.005 | 0.400-2.526 | 0.9909 |
| ASGSH | 1.137 | 0.442-2.920 | 0.0707 |
| Solid component ≥ 40% | 4.033 | 1.435-11.333 | 0.0082 |
| Metatypical pattern | 1.412 | 0.496-4.024 | 0.5180 |
| Lymphovascular invasion | 1.761 | 0.663-4.672 | 0.2560 |
| Perineural invasion | 0.636 | 0.238-1.705 | 0.3689 |
| Bone invasion | 1.108 | 0.364-3.372 | 0.8561 |
